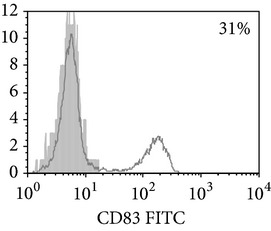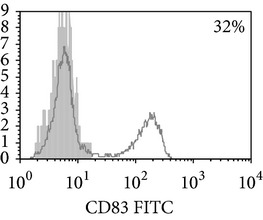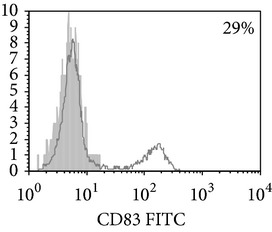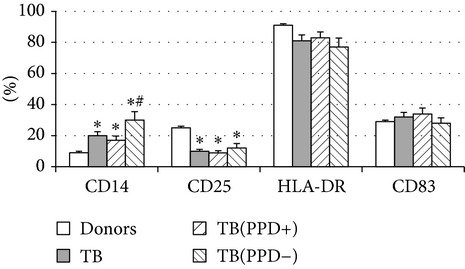# Impairments of Antigen-Presenting Cells in Pulmonary Tuberculosis

**DOI:** 10.1155/2015/793292

**Published:** 2015-08-03

**Authors:** Ludmila V. Sakhno, Ekaterina Ya. Shevela, Marina A. Tikhonova, Sergey D. Nikonov, Alexandr A. Ostanin, Elena R. Chernykh

**Affiliations:** ^1^Research Institute of Clinical Immunology, Russian Academy of Medical Sciences (RAMS), Siberian Branch (SB), Yadrintsevskaya Street 14, Novosibirsk 630099, Russia; ^2^Novosibirsk Tuberculosis Clinical Hospital No. 1, Vavilova Street 14, Novosibirsk 630082, Russia

## Abstract

The phenotype and functional properties of antigen-presenting cells (APC), that is, circulating monocytes and generated *in vitro* macrophages and dendritic cells, were investigated in the patients with pulmonary tuberculosis (TB) differing in lymphocyte reactivity to *M. tuberculosis* antigens (PPD-reactive versus PPD-anergic patients). We revealed the distinct impairments in patient APC functions. For example, the monocyte dysfunctions were displayed by low CD86 and HLA-DR expression, 2-fold increase in CD14^+^CD16^+^ expression, the high numbers of IL-10-producing cells, and enhanced IL-10 and IL-6 production upon LPS-stimulation. The macrophages which were *in vitro* generated from peripheral blood monocytes under GM-CSF were characterized by Th1/Th2-balance shifting (downproduction of IFN-*γ* coupled with upproduction of IL-10) and by reducing of allostimulatory activity in mixed lymphocyte culture. The dendritic cells (generated *in vitro* from peripheral blood monocytes upon GM-CSF + IFN-*α*) were characterized by impaired maturation/activation, a lower level of IFN-*γ* production in conjunction with an enhanced capacity to produce IL-10 and IL-6, and a profound reduction of allostimulatory activity. The APC dysfunctions were found to be most prominent in PPD-anergic patients. The possible role of APC impairments in reducing the antigen-specific T-cell response to *M. tuberculosis* was discussed.

## 1. Introduction

The immune response against* M. tuberculosis* (*Mtb*) is a complex process which involves many components of immune system. Professional antigen-presenting cells (APCs), including monocytes/macrophages and dendritic cells (DCs), play a major role in generating a protective immune response against* Mtb* by presenting antigens to T cells, recruiting immune cells at the site of infection, and directing T-cell response [1–3]. Therefore, functional impairments of APCs are considered to be an important mechanism of immune escape leading to* Mtb* persistence. Defective functions of APCs can be caused by a direct effect of* Mtb* on expression of surface molecules and production of cytokines by infected macrophages and DCs [4, 5].* Mtb* impairs DC maturation, reduces their ability to present mycobacterial antigens and to stimulate specific CD4^+^ T cells, inhibits secretion of IL-12 by DCs, and increases production of IL-10 which is able to suppress T-cell response and migration of DCs to draining lymph nodes [6–10]. Interaction of macrophages with pathogen causes pronounced alterations of phagosome function and suppresses their antigen-presenting function through the inhibition of synthesis and expression of MHC class II molecules [11–14].

Importantly, blood monocytes represent an important source of APCs capable of migrating to the infected site and differentiating into macrophages and DCs. Despite the absence of direct infection of circulating monocytes with* Mtb* many studies reported an altered phenotype and functions of monocytes in pulmonary tuberculosis [15–17]. Given an important role of monocytes as precursors of DCs and macrophages, their dysfunctions can result in pronounced impairments of monocyte-derived DCs (MDDC) and monocyte-derived macrophages (MDM).

In the present study we investigated whether blood monocytes, MDM and MDDC obtained from TB patients and healthy donors, differed in any significant way. Besides we attempted to clarify whether impairments of antigen-presenting function and cytokine secretion are similar in different APC types and how they are related to the defect of the antigen specific T-cell response in pulmonary tuberculosis. As T-cell impairments in pulmonary TB patients are manifested* in vitro* by downregulation of proliferative activity and/or production of IFN-*γ* in response to tuberculin purified protein derivative (PPD) [6, 18], the comparative analysis of APCs was conducted not only between TB patients and healthy subjects, but also between PPD-anergic and PPD-reactive patients.

## 2. Materials and Methods

### 2.1. Patients

The patients with active pulmonary tuberculosis (TB) were recruited from Novosibirsk Tuberculosis Clinical Hospital No. 1. The study involved 192 patients with pulmonary TB (125 males and 67 females aged from 20 to 64 years) including 68 with fibrocavernous, 100 with infiltrative, and 24 with disseminated TB. Positive for* M. tuberculosis* sputum specimens were revealed in 123 patients. Multidrug resistance (MDR) was registered in 69 patients. The TB patients underwent the standard antimicrobial treatment, including first-line drugs (combination of tubazid, rifampicin, streptomycin, ethambutol, and pyrazinamide) and in patients with MDR the second-line drugs (the combination of fluoroquinolones with amikacin or kanamycin, capriomycin, and cycloserine). The control group included 90 sex- and age-matched healthy subjects. The signed informed consent was obtained before the examination from all the patients.

### 2.2. Isolation of Cells and Evaluation of Proliferative Response

Mononuclear cells (MNCs) were isolated from heparinized venous blood by Ficoll-Verographin density gradient centrifugation and cultivated in 96-well plates (0.1 × 10^6^ per well) in RPMI-1640 (Sigma-Aldrich, USA) medium, completed with 0.3 mg/mL L-glutamine, 5 mM HEPES buffer, 100 *μ*g/mL gentamycin, and 10% inactivated human AB serum. In order to stimulate cell proliferative response, tuberculin-purified protein derivative (PPD) was used in a dose of 50 *μ*g/mL. Proliferation intensity was evaluated on the 6th day based on ^3^H-thymidine incorporation (1 *μ*Ci per well), adding 18 hours before the end of cultivation. Depending on proliferative response level, patients were divided into 2 subgroups: those with retained (>12,500 cpm; PPD-reactive TB patients) and those with reduced (<12,500 cpm; PPD-anergic TB patients) response to PPD.

### 2.3. Isolation of Monocytes and Generation of Monocyte-Derived Macrophages and Monocyte-Derived Dendritic Cells

Monocytes (Mo) were isolated in 6-well plates (Nuclon, Denmark) by adhesion of MNCs (3 × 10^6^ cells/mL) to the plastic in the presence of 5% human AB serum. Monocyte-derived macrophages (MDM) were generated by culturing adherent fraction of MNCs during 7 days in RPMI-1640 medium completed with 5% autoplasma, 2% fetal calf serum (FCS, Biolot, Russia), 2-mercaptoethanol (5 × 10^−5^ M, Serva, Germany), pyruvate Na (2 × 10^−3^ M, Sigma-Aldrich, USA), and 1% nonessential amino acid solution in the presence of GM-CSF (50 ng/mL, Sigma-Aldrich, USA). In 7 days macrophages were harvested using 0.25% trypsin/EDTA solution. Monocyte-derived dendritic cells (MDDC) were generated by culturing adherent fraction of MNCs during 4 days in RPMI-1640 medium with 5% FCS in the presence of GM-CSF (40 ng/mL) and IFN-*α* (1,000 U/mL, Roferon-A, Roche, Switzerland), followed by maturation over 24 hours in the presence of 10 *μ*g/mL lipopolysaccharide (LPS* E. coli* 0111:B4, Sigma-Aldrich, USA).

### 2.4. Phenotypic Analysis of Mo, MDM, and MDDC

Evaluation of surface markers expression on antigen-presenting cells was conducted with phycoerythrin- (PE-) labeled monoclonal anti-CD14 antibodies and FITC-labeled anti-CD16, CD25, CD83, CD86, and HLA-DR (PharMingen, USA) using flow cytofluorometry (FASC Calibur, Becton Dickinson, USA). To evaluate CD14^+^CD16^+^ cells, Mo were incubated with FITC-labeled anti-CD16 and PE-labeled anti-CD14 antibodies and then two-color cytometry analysis was conducted.

### 2.5. Intracellular Cytokine Assay

The estimation of intracellular expression of TNF-*α* and IL-10 in CD14^+^ Mo was performed by flow cytometry assays using permeabilization of cells. The number of cells with intracellular expression of TNF-*α* or IL-10 was estimated in monocytic gate using PerCP-labeled anti-CD14, FITC-labeled anti-TNF-*α*, and PE-labeled anti-IL-10 antibodies (Becton Dickinson).

### 2.6. The Estimation of Cytokine-Secreting Activity of Mo, MDM, and MDDC

The cytokines were assessed in 7-day MDM culture supernatants and in 5-day MDDC culture supernatants which were collected and stored at −80°C until measurement. The concentrations of TNF-*α*, IFN-*γ*, IL-6, IL-10, and IL-18 were evaluated by commercial ELISA kits (Vector-Best, Russia). The production of IL-6 and IL-10 was measured in Mo cultures which were harvested, washed, and then cultivated for additional 48 h with or without LPS (10 *μ*g/mL).

### 2.7. Evaluation of Allostimulatory Activity of MDM and MDDC

Allostimulatory activity of MDM and MDDC was evaluated in mixed lymphocyte culture (MLC) after cultivation of donor MNCs (0.1 × 10^6^/well) in round-bottom 96-well plates in the presence of allogeneic antigen-presenting cells from donors or TB patients in the ratio 10 : 1. Proliferation intensity was evaluated using radiometry on the 5th day based on ^3^H-thymidine incorporation.

### 2.8. Statistical Analysis

Statistical analysis was carried out using software package “Statistica 6.0.” To reveal significant difference of values compared, nonparametric Mann-Whitney* U* test was employed. The level of *P* < 0.05 was considered significant. Spearman rank correlation was used to investigate relationships between characteristics.

## 3. Results

**Figure 1:**
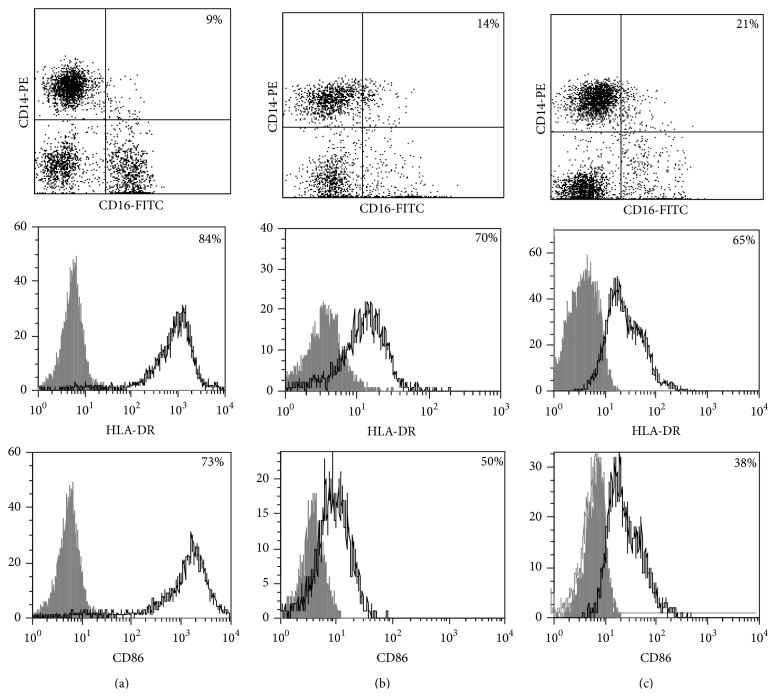
Surface antigen expression on circulating monocytes obtained from peripheral blood of TB patients (a), PPD-reactive (b) and PPD-anergic (c) TB patients. Open histogram represents stained cells (patient Mo) and the filled histogram represents isotype specific control.

Phenotypic analysis of freshly isolated monocytes (Table 1, Figure 1) revealed that monocytes from TB patients had a lower number of HLA-DR^+^ and CD86^+^ cells. Monocytes obtained from both PPD-reactive and PPD-anergic patients showed a decrease in HLA-DR and CD86 expression. Besides, TB patients demonstrated a significant increase in CD14^+^CD16^+^ monocytes, the level of which on average twice exceeded that of healthy subjects. The most pronounced increase in CD14^+^CD16^+^ monocytes was revealed in the PPD-anergic patients. The elevated rate (>17%) of CD14^+^CD16^+^ cells in this group (62%, 16/26) was observed twice oftener than among patients with the undiminished proliferative MNC response to PPD (26%, 10/39, *P*_TMΦ_ = 0.04).

**Figure 2:**
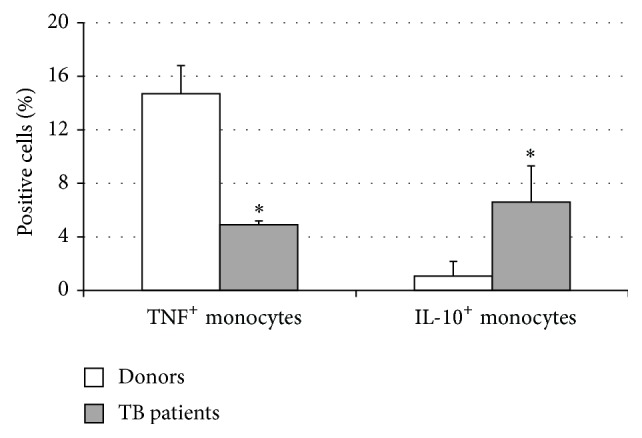
The spontaneous intracellular expression of TNF-*α* and IL-10 in donor (*n* = 8) and TB patient (*n* = 8) circulating CD14^+^ monocytes. The data are presented as M ± S.E. ^*^ *P*_*U*_ < 0.05 (Mann-Whitney* U*-criterion) between healthy donors and TB patients.

An evaluation of intracellular cytokine expression showed that monocytes from TB patients were characterized by a 3-fold decrease of TNF-*α*-secreting cells and a 6-fold increase of IL-10 secreting cells as compared to monocytes from healthy subjects (Figure 2). What is more, there appeared to exist an inverse correlation (*r*_*S*_ = −0.62, *P*_*S*_ < 0.01; *n* = 19) between the numbers of CD14^+^CD16^+^ cells and TNF-*α* ^+^ monocytes.

To ascertain whether an increase in IL-10^+^ monocytes was accompanied by an increased production of immunosuppressive/anti-inflammatory cytokines we additionally evaluated the production of IL-10 and IL-6 in 48-hour monocyte cultures. Concentrations of IL-6 and IL-10 in LPS-stimulated supernatants from patient monocyte cultures (Table 2) were significantly higher than in donor cultures. Notably, the most pronounced increase of LPS-induced production of IL-6 and IL-10 was registered in PPD-anergic patients. The obtained data showed TB infection is associated with a decrease in proinflammatory and an increase in anti-inflammatory/immunosuppressive activity which resulted in suppressing of PPD-response.

Considering the monocyte dysfunctions in TB patients, we further investigated the monocyte-derived macrophages (MDM). MDM viability on 7th day was no lower than 90% in all experiments. MDM yield was found to be similar in donors and TB patients (29,000 ± 4,000 and 31,000 ± 5,000/10^6^ MNC, resp.). Phenotypic analysis (Table 3) showed that both donor and patient MDM were highly positive (about 80%) for CD 14. MDM from TB patients with PPD-anergy displayed a significant decrease of HLA-DR^+^ and CD86^+^ cells, whereas the expression of these molecules on MDM from PPD-reactive patients did not differ from that of donors. In contrast to monocytes, an increased number of MDM expressing CD16 (about 50%) was found in patients with the intact response to PPD that was significantly higher than in donors and patients with lowered PPD-induced proliferative response. These data mean the regulatory functions of CD16^+^ monocytes and CD16^+^MDM could differ. Thus, MDM from patients with an adequate antigen-specific response do not differ in expression of antigen-presenting and costimulatory molecules from donor MDM while MDM from PPD-anergic patients show a lowered expression of molecules necessary for an effective antigen presentation and T-lymphocyte costimulation.

An important function of MDM during immune response is cytokine production. An analysis of cytokine concentration in 7-day patient MDM cultures did not reveal a significant difference in the TNF-*α*, IL-6, and IL-18 levels as compared to healthy individuals. At the same time MDM from PPD-reactive and PPD-anergic patients displayed a 10-fold decrease of IFN-*γ* production. However, concentration of IL-10 in MDM cultures from PPD-anergic patients was 3 times higher than in MDM cultures from healthy subjects and PPD-reactive patients. These results testified to a higher MDM immunosuppressing potential in patients with decreased PPD response.

An evaluation of MDM allostimulatory activity in a mixed lymphocyte culture revealed that macrophages from PPD-reactive patients, similar to donor MDM, effectively stimulated the proliferation of allogeneic MNC (Table 4).

At the same time a proliferative response of alloantigen-stimulated T-lymphocytes induced by MDM from PPD-anergic patients was almost 6 times lower as compared to donors (*P*_*U*_ < 0.05). Thus, the lowered expression of antigen-presenting and costimulatory molecules and the increased level of IL-10 production by MDM obtained from PPD-anergic patients were associated with a decreased macrophage ability to stimulate T-cell proliferation in a mixed lymphocyte culture.

Thereafter, we compared phenotypic and functional properties of monocyte-derived dendritic cells (MDDC) in TB patients and healthy individuals. Donor and patient MDDC yield did not differ and comprised accordingly 27,000 ± 7,000 and 35,000 ± 6,000/10^6^ MNC. In addition, donor and patient MDDC did not vary in the number of HLA-DR^+^ and CD83^+^ cells (Table 5). Nevertheless, patient MDDC were characterized by an increased level of CD14^+^ cells more pronounced in PPD-anergic patients and a decreased level of CD25^+^ cells registered in patients with both a lowered and an intact proliferative response of MNC to PPD.

Evaluation of cytokine levels in 5-day DC cultures showed that patient MDDC were characterized by an increased production of IL-6 and Il-10 and highly decreased level of IFN-*γ* (Table 4). It is typical that IL-10 production in MDDC cultures from PPD-anergic patients was significantly higher than in MDDC cultures from PPD-reactive patients. When investigating MDDC allostimulatory activity, we discovered that patient MDDC possessed an impaired ability to stimulate T-cell proliferative response in mixed lymphocyte cultures (Table 4). The most pronounced defect of MDDC allostimulatory activity was found in PPD-anergic patients.

## 4. Discussion

Our data conclusively show that in TB patients phenotypic and functional disorders are typical both for circulating monocytes and for MDM and MDDC. Our results displaying a decrease in HLA-DR^+^ and CD86^+^ monocytes and an increase in CD14^+^CD16^+^ cells in pulmonary tuberculosis confirm the results of other investigators [19, 20]. Balboa et al. showed that CD14^+^CD16^+^ cell functions could significantly differ depending on their localization [17]. For example, CD16^+^ monocytes in pleural effusion represent effective APCs since these monocytes express receptors for* Mtb* recognition and antigen presentation (DC-SIGN, MR, CD11b, and CD1b). At the same time increase in peripheral blood CD16^+^ monocytes is associated with the severity of pulmonary TB. In this respect our data showing a more pronounced augmentation of CD14^+^CD16^+^ monocytes in PPD-anergic patients which differ by higher severity [18] is still another argument of an unfavorable prognostic role of CD16^+^ monocytes in pulmonary tuberculosis. We should note that CD14^+^CD16^+^ monocytes are characterized by an increased proinflammatory activity [21] and according to Balboa the number of these cells in circulation correlates with TNF-*α* level in blood plasma [17]. Nevertheless, we found an inverse correlation (*r*_*S*_ = −0.62, *P* < 0.01) between the number of CD14^+^CD16^+^ cells and a percentage of monocytes with an intracellular TNF-*α* expression. Earlier we showed that the number of IL-10^+^ cells within CD16^+^ population is significantly higher in TB patients than in healthy subjects [15]. These data together with our present results showing that the most pronounced increase of CD14^+^CD16^+^ cells in PPD-anergic patients was associated with the highest increase of IL-10 can testify to a high monocyte suppressive activity and its crucial role in suppressing an antigen specific response.

We should note that monocyte alterations were found in TB patients regardless of the level of antigen specific response though some parameters (i.e., the number of CD14^+^CD16^+^ cells, IL-6 and IL-10 production) were more pronounced in PPD-anergic patients. At the same time an impaired macrophage function (i.e., a decreased number of CD86^+^ and HLA-DR^+^ cells, an increased IL-10 production, and a decreased ability to stimulate allogeneic T-cell proliferation) was only typical for MDM from PPD-anergic patients.

The main approach to study macrophages during TB in humans is an analysis of how* Mtb* interferes with these cells. MDM generated in the presence of* Mtb* were previously observed to have decreased MHC class II, CD68, CD86, and CD36 expression [16]. Additionally,* Mtb*-infected monocyte-derived macrophages were found to produce the immunosuppressive cytokine IL-10 which inhibited IL-12 secretion [2]. Nagabhushanam et al. showed that IL-6 secreted by* Mtb*-infected macrophages inhibits the responses to IFN-*γ* [12], thus limiting the ability of IFN-*γ* to stimulate macrophages to kill* Mtb*. In the present study we were the first to describe the properties of monocyte-derived macrophages from active TB patients and to show that an impairment of monocyte-derived macrophages is similar to the impairment observed upon infecting macrophages with* Mtb*.

In contrast to MDM, MDDC dysfunction was found in both PPD-anergic and PPD-reactive patients, but it was more pronounced in patients with a decreased PPD response. Earlier Rajashree et al. showed that TB patient MDDC generated with GM-CSF and IL-4 are characterized by downregulation of CD1a, MHC class II, CD80, and CD83 expression and impaired allostimulatory activity [22]. In turn, Balboa et al. showed that the impairment of DC maturation in GM-CSF and IL-4 cultures was caused by a high content of CD16^+^ monocytes [23] which differentiated into a CD1a^−^DC-SIGN^low^ population characterized by a poor mycobacterial Ag-presenting capacity. In our study we generated MDDC with GM-CSF and IFN-*α*. This type of DCs has a number of phenotypic and functional differences from IL-4-derived DCs [24–26]. At the same time, a typical feature of MDDC in our study was a decreased ability to stimulate allogeneic T-cell proliferation. Thus, DC impairments were revealed not only in IL-4-derived DCs but also in IFN-derived cells.

A common MDM/MMDC functional defect is an impairment of their secretory activity (IFN-*γ* production deficit in couple with an increased IL-10 secreting activity) and a decreased ability to stimulate allogeneic T-cell proliferation. The cell-mediated immune response is known to be critical in the host defense against* Mtb*. Activated T helper 1 (Th1) lymphocytes play an important role in granuloma formation and through production of IFN-*γ* stimulate the antimicrobial activity of infected macrophages, allowing intracellular bacterial killing. In contrast, IL-10 inhibits antimicrobial effector mechanisms, the expression of costimulatory molecules, and the production of proinflammatory cytokines by APCs [27]. It is well known that IL-6 can induce naïve CD4^+^ T-lymphocyte polarization toward the Th2, and IL-10 is an inhibitor of Th1 cells [2, 27]. In this respect the imbalance of IFN-*γ*/IL-10, IL-6 production typical for APCs in TB patients is apparently a cause of low antigen-specific response in PPD-anergic patients. As an additional mechanism of PPD-anergy in TB, we can consider a decreased expression of antigen-presenting and costimulatory molecules in APCs which causes an impairment of their antigen-presenting function. Indeed, we found the most expressed inhibition of macrophage and DC allostimulatory activity in PPD-anergic patients.

## 5. Conclusions

The phenotype and functional properties of antigen-presenting cells, that is, circulating monocytes and* in vitro* generated macrophages and dendritic cells, are altered in TB patients. These impairments are most pronounced in PPD-anergic patients and may be the cause of low antigen-specific T-cell response.

## Figures and Tables

**Table 1:** The phenotypic characteristics of monocytes obtained from peripheral blood of healthy donors and TB patients.

Markers (%)	Healthy donors	TB patients		
		All patients	PPD-reactive patients	PPD-anergic patients
CD14^+^CD16^+^	8.9 ± 1.2 (15)	18.0 ± 1.2 (65)^*^	15.4 ± 1.0 (39)^*^	21.4 ± 2.4 (26)^∗#^
HLA-DR^+^	84.1 ± 1.5 (10)	69.7 ± 1.8 (72)^*^	69.8 ± 2.2 (50)^*^	69.1 ± 3.7 (22)^*^
CD86^+^	66.1 ± 4.5 (9)	48.3 ± 4.9 (18)^*^	48.6 ± 5.4 (12)^*^	40.5 ± 4.6 (6)^*^

The relative numbers of Mo (M ± S.E.) expressing different markers were presented in healthy donors and TB patients (the whole group), including PPD-reactive and PPD-anergic TB patients. The number of cases is indicated in parentheses. ^*^ *P*_*U*_ < 0.05 (Mann-Whitney *U*-criterion) with healthy donors; ^#^ *P*_*U*_ < 0.05 between PPD-reactive and PPD-anergic TB patients.

**Table 2:** The production of cytokines by Mo obtained from peripheral blood of healthy donors and TB patients.

Cytokine (pg/mL)	Stimulator	Healthy donors (*n* = 8)	TB patients		
			All patients (*n* = 17)	PPD-reactive patients (*n* = 8)	PPD-anergic patients (*n* = 9)
IL-6	0	1,509 ± 781	2,995 ± 390	2,748 ± 756	3,206 ± 374
	LPS	1,902 ± 720	4,015 ± 315^*^	3,596 ± 708	4,329 ± 146^*^

IL-10	0	27 ± 17	59 ± 12.8	59 ± 19.4	58 ± 18.1
	LPS	70 ± 30.9	201 ± 44.7^*^	166 ± 65.5	233 ± 62.8^*^

The average values (M ± S.E.) of spontaneous (0) and LPS-stimulated (LPS) cytokine production in 48-hour cultures of monocytes (10^5^/per well) obtained from peripheral blood of healthy individuals and TB patients were presented. ^*^ *P*_*U*_ < 0.05 (Mann-Whitney *U*-criterion) with healthy donors.

**Table 3:** Surface antigen expression on monocyte-derived macrophages from TB patients (A), PPD-reactive (B) and PPD-anergic (C) TB patients. (I) Open histogram represents stained cells and the filled histogram represents isotype specific control. (II) The number of CD14^+^, CD16^+^, HLA-DR^+^, and CD86^+^ MDM is presented as M ± S.E. ^*^ *P*_*U*_ < 0.05 (Mann-Whitney *U*-criterion) with healthy donors; ^#^ *P*_*U*_ < 0.05 between PPD-reactive and PPD-anergic TB patients.

MDM	(A)	(B)	(C)
(I)			
CD14	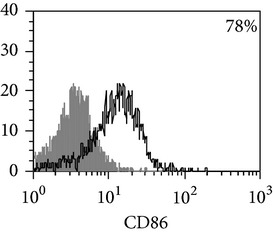	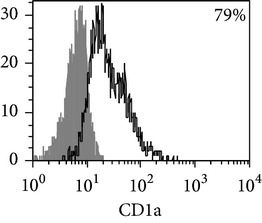	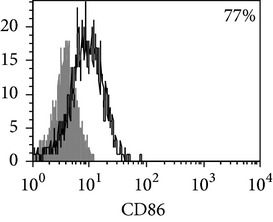
CD16	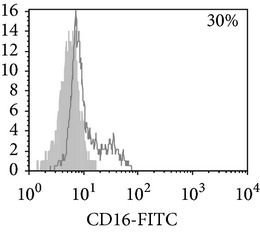	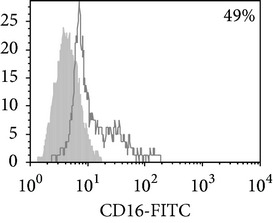	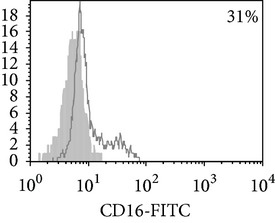
HLA-DR	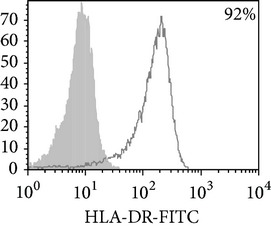	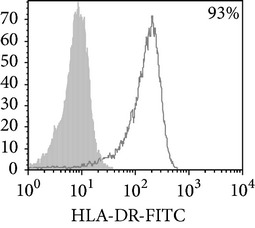	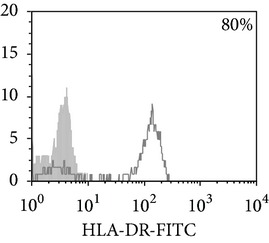
CD86	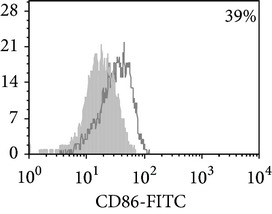	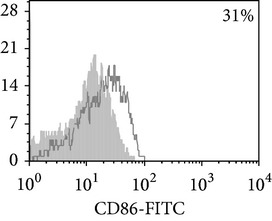	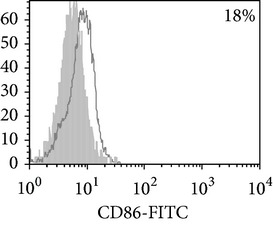
(II)	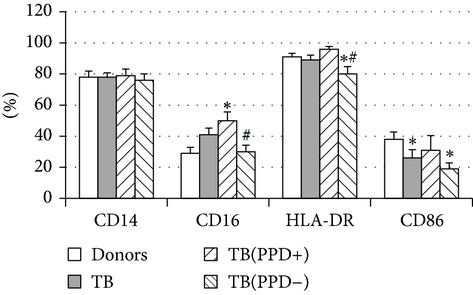		

**Table 4:** Secretory and allostimulatory activities of MDM and MDDC. The data are presented as M ± S.E. ^*^ *P*_*U*_ < 0.05 (Mann-Whitney *U*-criterion) with healthy subjects; ^#^ *P*_*U*_ < 0.05 between PPD-reactive and PPD-anergic TB patients.

MDM	MDDC
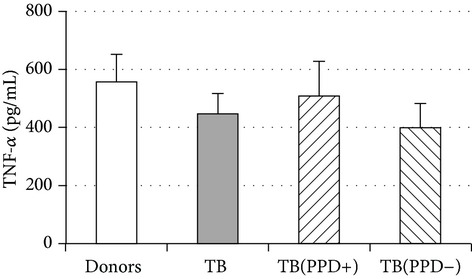	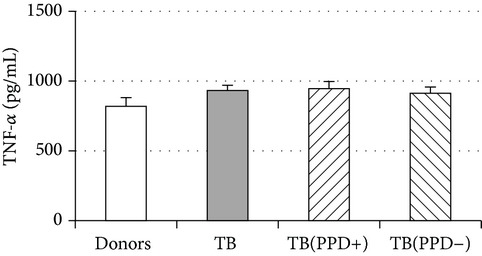
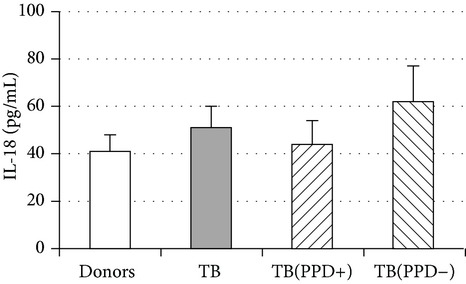	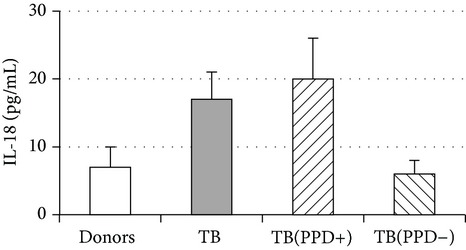
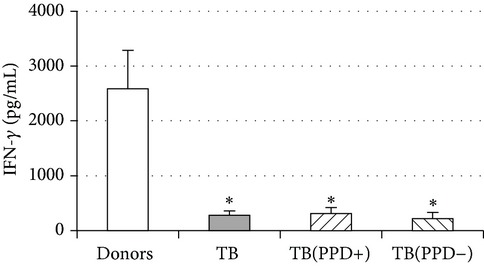	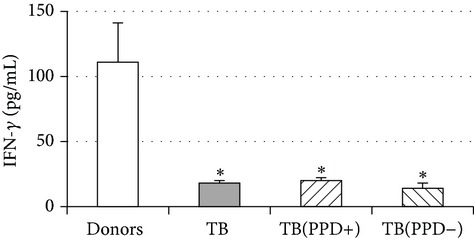
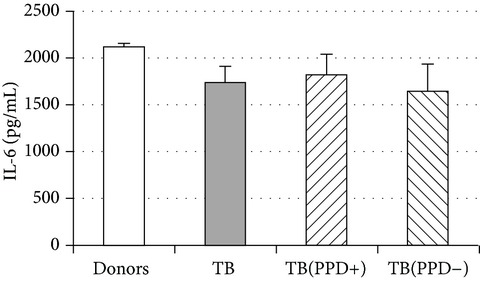	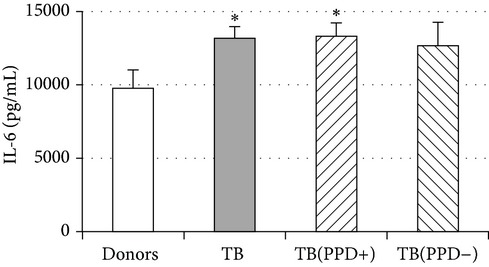
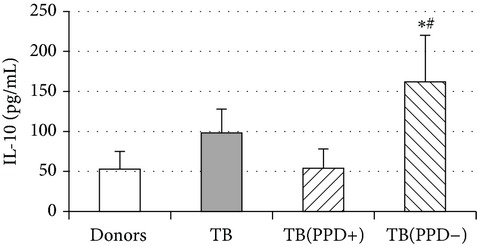	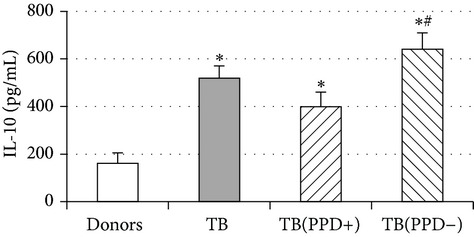
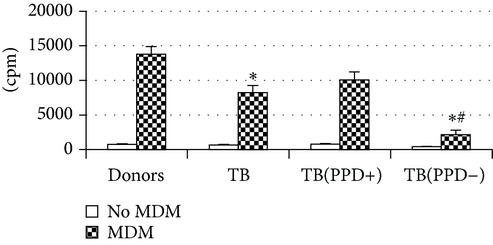	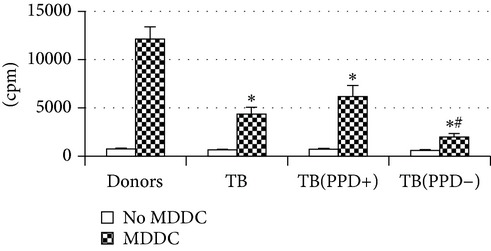

**Table 5:** Surface antigen expression on monocyte-derived DCs from TB patients (A), PPD-reactive (B) and PPD-anergic (C) TB patients. (I) Open histogram represents stained cells and the filled histogram represents isotype specific control. (II) The number of CD14^+^, CD25^+^, HLA-DR^+^, and CD83^+^ MDDC is presented as M ± S.E. ^*^ *P*_*U*_ < 0.05 (Mann-Whitney *U*-criterion) with healthy subjects; ^#^ *P*_*U*_ < 0.05 between PPD-reactive and PPD-anergic TB patients.

MDDC	A	B	C
CD14	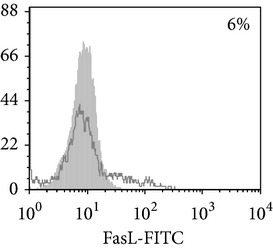	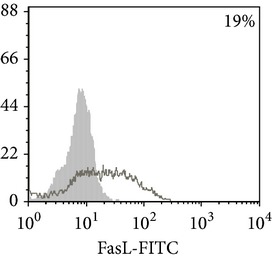	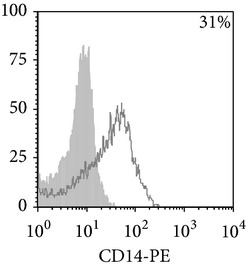
CD25	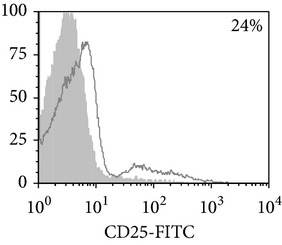	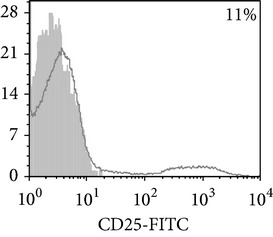	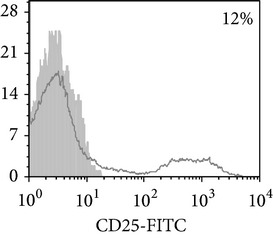
HLA-DR	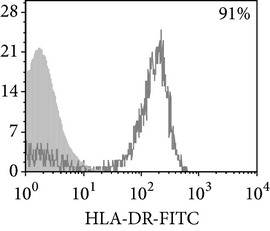	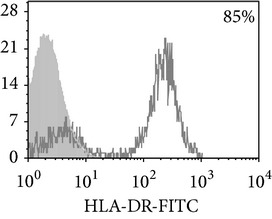	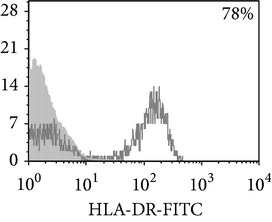
CD83